# Carbon Dots as Multifunctional Nanomaterials: A Review on Antimicrobial Activities and Fluorescence-Based Microbial Detection

**DOI:** 10.3390/molecules30193969

**Published:** 2025-10-03

**Authors:** Andreas Romulo, Steven Suryoprabowo, Raden Haryo Bimo Setiarto, Yahui Guo

**Affiliations:** 1 Food Technology Department, Faculty of Engineering, Bina Nusantara University, Jakarta 11480, Indonesia; steven.suryo@binus.ac.id; 2 Research Center for Applied Microbiology, National Research and Innovation Agency (BRIN), Kawasan Sains dan Teknologi (KST) Soekarno, Jl. Raya Jakarta-Bogor KM. 46, Cibinong, Bogor 16911, Indonesia; rhar002@brin.go.id; 3 School of Food Science and Technology, Jiangnan University, No. 1800 Lihu Avenue, Wuxi 214122, China; guoyahui@jiangnan.edu.cn

**Keywords:** carbon dots (CDs), antimicrobial resistance, fluorescence-based detection, reactive oxygen species (ROS)

## Abstract

The increasing prevalence of antimicrobial resistance and the persistent challenge of infectious diseases highlight the critical necessity for novel approaches that integrate pathogen management with swift detection methods. Carbon dots (CDs) are a versatile class of fluorescent nanomaterials that have garnered increasing attention owing to their tunable surface chemistry, strong photoluminescence, high stability, and biocompatibility. Recent studies demonstrate that CDs possess broad-spectrum antibacterial and antifungal activities via multiple mechanisms, including the generation of reactive oxygen species, disruption of membranes, inhibition of biofilms, and synergistic interactions with conventional antimicrobials. The performance is significantly affected by precursor selection, heteroatom doping, and surface functionalization, with minimum inhibitory concentrations reported to range from highly potent at the microgram level to moderate at elevated concentrations. The intrinsic fluorescence of CDs, in addition to their antimicrobial activity, facilitates their use as sensitive and selective probes for microbial detection, allowing for rapid and real-time monitoring in biomedical, food safety, and environmental settings. This review summarizes recent advancements in the antimicrobial properties of carbon dots (CDs) and their fluorescence-based applications in microbial detection. It emphasizes their theranostic potential and future prospects as multifunctional nanomaterials for combating infectious diseases and ensuring microbial safety.

## 1. Introduction

Pathogenic microorganisms play a critical role in the global spread of infectious diseases, contribute substantially to morbidity and mortality rates and place a heavy burden on healthcare systems worldwide, especially in low- and middle-income countries, where access to timely and effective treatment remains limited [1]. Moreover, the global health landscape is further complicated by the emergence of new, previously unrecognized, or even re-emerging infectious agents that have the potential to cause widespread epidemics. Emerging infectious diseases include new illnesses like COVID-19 and familiar ones like influenza and tuberculosis that are spreading more quickly or appearing again in certain regions [2,3]. What is particularly concerning is bacterial co-infections, which were identified in up to 16% of critically ill patients, further complicating treatment and increasing the risk of severe outcomes [4]. Antibiotics are indeed the most prescribed medications for the treatment of bacterial infections. However, the growing resistance to existing antibiotics, coupled with the slow pace of new antibiotic development, underscores the urgent need to explore and develop alternative antimicrobial agents for effective treatment [5,6].

The integration of nanotechnology and microbiology has created new opportunities for addressing bacterial infections and shows significant promise [7]. Recent advancements in nanotechnology have facilitated significant progress in the synthesis and application of nanomaterials, leading to the creation of highly engineered structures with distinct physicochemical properties. Nanomaterials such as metal nanoparticles, metal oxides, and carbon-based nanostructures have been thoroughly investigated for their antimicrobial properties [8]. Among these materials, carbon dots (CDs) have garnered significant research interest for their antimicrobial properties, attributed to their advantageous physicochemical characteristics and functional versatility.

CDs are a new and versatile category of zero-dimensional carbon-based nanomaterials, generally measuring less than 10 nm in diameter, characterized by their inherent photoluminescence and diverse surface chemistry [9]. Following their unexpected discovery in 2004 during the purification of single-walled carbon nanotubes and subsequent formal characterization in 2006 [10], they have attracted significant attention across multiple scientific fields, including optoelectronics, bioimaging, drug delivery, and antimicrobial therapy, owing to their remarkable physicochemical and biological properties [11]. CDs function as antimicrobials mainly via photodynamic and, in certain instances, photothermal mechanisms, which are facilitated by their photosensitizing characteristics [12]. Their ability to generate reactive oxygen species (ROS) under light irradiation leads to oxidative damage in microbial cells [13]. This antimicrobial activity is strongly influenced by the surface functional groups of CDs, which affect solubility, surface charge, membrane interactions, and ROS generation [14]. Additionally, these same surface properties allow CDs to function as probes for microbial detection. Functionalized carbon dots can selectively adhere to bacterial cells and demonstrate fluorescence alterations upon engagement, facilitating real-time pathogen identification. In some instances, CDs provide dual functionality, initially identifying bacteria through fluorescence modulation, subsequently eradicating them through light-activated antimicrobial mechanisms. This theranostic potential establishes CDs as valuable instruments for microbial detection and regulation, with extensive applications in biomedical diagnostics, food safety, and environmental surveillance.

The intrinsic fluorescence of carbon dots, alongside their antimicrobial functions, presents considerable potential for microbial detection. Carbon dots exhibit tunable emission, surface functionalization, and biocompatibility, allowing them to function as sensitive probes for rapid pathogen identification while concurrently delivering therapeutic effects via light-activated mechanisms. The dual functionality of CDs establishes them as promising theranostic agents, integrating antimicrobial activity with real-time diagnostic capabilities. This review highlights the antimicrobial efficacy of CDs and emphasizes their emerging role in fluorescence-based microbial detection.

## 2. Antimicrobial Mechanism Expressed by Carbon Dots (CDs)

The disruption of microbial cell structural integrity is one of the most widely recognized and effective antibacterial approaches [15]. In order to ensure their continued existence, both bacteria and fungi possess structural components that are vital to their life. Bacteria, which are unicellular prokaryotes, have essential components such as the cell wall, plasma membrane, cytoplasm, nucleoid region, and a variety of structural proteins [16]. On the other hand, fungi, which are eukaryotic microorganisms, have more complex structures, such as a cell wall that is rich in chitin, a cell membrane that contains ergosterol, organelles, and a nucleus that is clearly defined [17]. CDs, which are characterized by their extremely small size, high surface-area-to-volume ratio, and varied surface functions, have demonstrated the capability to interact with and disturb the critical structures of microorganisms.

The antibacterial efficiency of carbon dots (CDs) is closely associated with their structural attributes, such as particle size, surface charge, functional groups, passivation layers, and elemental doping [18]. Particle size is a crucial determinant among these factors. CDs smaller than 5 nm demonstrate enhanced antibacterial efficacy owing to their capacity to intimately engage with and infiltrate microbial cell membranes. Previous research showed that smaller carbon dots (CDs) around 2 nm were more effective antibacterial agents than larger ones (~5 nm), particularly against *E. coli* and *S. aureus* [19]. Beyond size, the shape and curvature of CDs are critical for targeting. CDs engineered to mimic the Gaussian membrane curvature of specific bacteria, like spherical *S. aureus*, can physically bind and disrupt their membranes via curvature-induced stress [20]. A smaller particle possesses a higher surface area-to-volume ratio, so probably augmenting interactions with microbial surfaces and promoting intracellular penetration [21]. The surfaces of most bacterial cells possess a negative charge attributed to teichoic acids and lipopolysaccharides [20]. Consequently, positively charged CDs exhibit enhanced efficacy in adhering to bacterial membranes through electrostatic interactions. This results in heightened membrane permeability, efflux of intracellular substances, and eventually, cellular lysis. Research has shown that increasing the zeta potential of CDs enhances their antibacterial efficacy against both Gram-positive and Gram-negative bacteria [22,23]. Not all effective antimicrobial CDs possess a positive charge. Under specific conditions, negatively charged CDs may demonstrate antibacterial properties. Highly anionic CDs have demonstrated improved antibacterial efficacy under laser irradiation [24]. In this instance, van der Waals forces surpassed electrostatic repulsion, facilitating the adherence of the CDs to the bacterial surface, resulting in ROS generation and subsequent damage to proteins and the cell wall. However, in a separate study, negatively charged CDs showed antimicrobial activity mechanism through electrostatic repulsion, obstructing nutrient absorption and resulting in microbial isolation and mortality [25].

The antibacterial efficacy of CDs is also significantly affected by their surface functional groups, which govern their interaction with bacterial cells and establish the total surface charge of the particles. Amino (–NH_2_) groups and quaternary ammonium moieties, which possess a positive charge, facilitate robust electrostatic attraction to negatively charged bacterial membranes, resulting in membrane rupture and subsequent bacterial death [26]. Structural changes in CDs by doping heteroatoms into the carbon core or surface group also have been reported to generate antimicrobial effect. Nitrogen-doped carbon dots and polyethyleneimine-coated carbon dots have demonstrated improved antibacterial efficacy while minimizing harm to mammalian cells [27]. Through the doping process, carbon dots exhibit a synergistic antimicrobial mechanism that integrates multiple modes of bacterial inhibition such as surface charges modification and photodynamic activation.

Another antimicrobial mechanism of CDs was explained via a light-activated photodynamic (photosensitizer), which entails the production of reactive oxygen species (ROS) [25,28]. When exposed to visible or natural light, CDs become photoexcited and transfer energy to adjacent oxygen molecules, resulting in the generation of ROS such as singlet oxygen (^1^O_2_) and hydroxyl radicals (**•**OH) [29]. Reactive species are pivotal in the inactivation of microbes. Specifically, the initial antimicrobial action involves the adhesion of CDs to the surface of bacterial cells, driven by electrostatic interactions between the positively charged CDs and the negatively charged bacterial cell walls, particularly in Gram-negative bacteria like *Escherichia coli* [30]. The process initiates with the adhesion of CDs to bacterial cell surfaces, mediated by electrostatic interactions. Upon activation by light, the resultant ROS target the bacterial cell membrane, leading to increased permeability and structural disruption. This damage enables reactive oxygen species (ROS) to infiltrate cells, inducing oxidative stress that affects essential biomolecules, including DNA, RNA, and proteins. Oxidative damage may impede gene expression, denature enzymes, and disrupt cellular functions, ultimately resulting in bacterial cell death via necrosis or programmed cell death [31]. Experimental studies have demonstrated that CDs exposed to light significantly reduce bacterial viability compared to dark conditions, underscoring the critical role of light in augmenting their bactericidal effects [32].

CDs function as antifungal agents by employing various mechanisms that compromise fungal cells, prevent biofilm formation, and disturbing the production of hyphae or yeast-transition [17,33]. Similarly to other positively charged nanoparticles, CDs with a positive surface charge demonstrate significant electrostatic attraction to the negatively charged fungal membrane, thereby enhancing cellular entry. Upon internalization, CDs can disturb the intracellular environment by hindering enzymatic functions, modifying ion concentrations, or exhibit strong photo-induced oxidative activity producing ROS such as singlet oxygen, mechanism notably linked to CDs enriched in electron-donating groups, including nitrogen-containing functionalities [34]. Disruptions can impair proteins and DNA, compromise membrane integrity, and result in the leakage of cytoplasmic contents [34,35]. Another antifungal strategy entails CDs to selectively bind to ergosterol, a crucial element of fungal membranes, thereby facilitating their attachment and traversal across the membrane barrier, leading to membrane disintegration and fungal cell death [36].

## 3. Antimicrobial Activities of Carbon Dots (CDs) Against Pathogenic Microorganisms

The swift emergence of antimicrobial resistance presents a significant risk to global public health, necessitating the urgent discovery and development of new antimicrobial drugs. Traditional antibiotics are progressively losing efficacy, resulting in enduring illnesses and elevated mortality rates. In this context, nanomaterials have emerged as viable alternatives, with carbon dots (CDs) receiving notable attention for their distinctive physicochemical properties, including adjustable surface functional groups, robust photoluminescence, high stability, and biocompatibility.

Antimicrobial susceptibility testing is an essential instrument for pharmacological development, epidemiological studies, and forecasting therapeutic results [37]. Evaluating the antimicrobial properties of CDs is essential as it assesses their capacity to suppress or eradicate pathogenic microorganisms, such as bacteria, fungus, and viruses. These investigations not only demonstrate their efficacy against clinically significant strains but also clarify mechanisms of action, including reactive oxygen species (ROS) production, membrane disruption, and biofilm inhibition [38]. Moreover, antimicrobial assays yield critical quantitative metrics, including minimum inhibitory concentration (MIC) and minimum bactericidal concentration (MBC), which are vital for comparing CDs with traditional antimicrobials and for informing their utilization in biomedical, food safety, and environmental domains.

Table 1 summarizes the results of antimicrobial activity of CDs from recent studies. There are three most common methodologies for evaluating the antimicrobial activity of CDs against several pathogenic microorganisms, namely disk diffusion, agar dilution, and broth microdilution.

The disk or agar diffusion method, often referred to as the Kirby–Bauer test, is a widely used qualitative or semi-quantitative technique for evaluating antimicrobial activity. In this method, a standardized microbial suspension is evenly spread over the surface of an agar plate to create a uniform lawn of growth. Filter paper disks impregnated with the test compound or alternatively wells cut into the agar and filled with the agent, are then placed on the surface. During incubation, the compound diffuses radially through the agar, interacting with the microorganisms. If the agent is effective, it inhibits microbial growth, producing a clear circular area around the disk or well known as the zone of inhibition [80].

Studies employing disk diffusion and agar-well diffusion methods demonstrated varying levels of antimicrobial activity among different carbon dots (CDs). The most pronounced inhibition zones were observed with *Taxus baccata*–derived CDs, which produced zones up to 33 mm [71], and castor seed N-CQD1, with a zone of 30 mm [73], indicating strong antimicrobial potency. Moderate inhibition was shown by spermidine-derived CDs (22 mm) [48] and *Prosopis juliflora* leaf CDs (16.7 mm) [76], while avocado peel CQDs exhibited smaller but consistent zones of 8–13 mm [54]. CDs synthesized from citric acid with β-alanine or ethylenediamine showed relatively weaker activity, with zones ranging between 10 and 12 mm [43,57]. L-arginine–based CDs, including their HPMC-modified form, displayed only minimal inhibition zones of 1–2 mm [78], suggesting limited effectiveness by comparison. Disk diffusion is the most widely used AST method in microbiology laboratories because of its low cost and ease of performance and applicability of numerous bacterial and fungal species [81]. Nevertheless, this method cannot be used to determine the minimum inhibitory concentration of CDs and its matrices due to unquantified amounts of CDs diffused to the agar medium, and should be treated only as the method to provide initial qualitative information on antimicrobial potency of CDs [80,81].

The dilution method is one of the most reliable and standardized techniques for evaluating antimicrobial activity, as it allows determination of the minimum inhibitory concentration (MIC) and, when necessary, the minimum bactericidal concentration (MBC) of a compound [82]. Agar dilution and broth dilution methods are closely associated with methods of determining the minimum inhibitory concentration (MIC), the minimal quantity of a chemical that inhibits observable bacterial or fungal proliferation. The differentiation between the two resides in the medium employed: agar dilution integrates the antimicrobial agent into solid agar plates, whereas broth dilution utilizes liquid broth tubes. In both methodologies, repeated dilutions of the antimicrobial agent are formulated, and a standardized inoculum of bacteria is introduced [80]. The MIC is determined by the presence or absence of microbial growth. These methodologies are crucial for identifying appropriate concentration of CDs that give potent antimicrobial activity and for monitoring changes in microbial susceptibility over time.

The antibacterial efficiency of carbon dots (CDs) is significantly affected by their production method, doping elements, and surface chemistry. A variety of CDs have been evaluated against different bacterial strains, demonstrating significant differences in the minimum inhibitory concentrations (MICs) and minimum bactericidal concentrations (MBCs). Glucose-derived carbon dots synthesized via a one-step hydrothermal technique (GCDs) and their boron-doped variants (BGCDs) exhibited minimum inhibitory concentrations (MIC) ranging from 312 to 625 µg/mL and minimum bactericidal concentrations (MBC) up to 1250 µg/mL against *Listeria monocytogenes* and *Escherichia coli* [40]. The elevated findings suggest that a substantial concentration is necessary for both inhibition and total bacterial elimination, potentially indicating restricted interaction with bacterial membranes or diminished generation of reactive oxygen species (ROS). In contrast, CDs modified or doped with heteroatoms exhibited improved antibacterial efficacy. Sulfur and nitrogen co-doped carbon dots (SN-CDs), synthesized from thiourea and o-phenylenediamine, demonstrated minimum inhibitory concentrations (MICs) as low as 16 µg/mL against both *E. coli* and *Staphylococcus aureus*, presumably attributable to enhanced surface activity and augmented electron transfer resulting in reactive oxygen species (ROS) formation. Phosphorus-doped carbon dots produced from glucose and phosphoric acid exhibited MIC values between 128 and 256 µg/mL against *E. coli*, *S. aureus*, and *Salmonella typhimurium* [60]. The findings indicate that heteroatom doping alters the surface charge and binding capability of carbon dots while also improving their capacity to infiltrate bacterial biofilms and membranes. Supplementary antibacterial studies from functionalized carbon dots, specifically those produced from L-histidine or guanidine [54,55,57], further corroborate the influence of precursor chemistry. These CDs demonstrated MIC values ranging from 64 to 256 µg/mL against both Gram-positive and Gram-negative bacteria, with MBCs exhibiting analogous patterns. Conversely, CDs derived from basic precursors as citric acid, urea, or acetic acid without further functionalization typically necessitated elevated MICs—reaching up to 1500 µg/mL [41,61,62,63,64,65,66].

Beyond MIC/MBC/MFC and inhibition zone measurements, several studies in the table evaluated antimicrobial efficacy of carbon dots (CDs) using viable cell counts and log reduction assays. For instance, arginine-based CDs and their composites (Arg-Ag, Arg-AgCl) not only produced inhibition zones but also demonstrated strong antimicrobial effects through CFU reduction, confirming their bactericidal activity beyond diffusion-based methods [44]. Similarly, polymer–CD composites such as PANI–CuO, PANI–TiO_2_, and PANI–SiO_2_ were tested using CFU counting, where PANI–CuO and PANI–TiO_2_ at 1 mg/mL significantly inhibited *Pseudomonas aeruginosa*, and PANI–TiO_2_ completely eradicated *Klebsiella pneumoniae* ATCC 700603, highlighting direct bactericidal effects verified by culture viability rather than zone inhibition [45]. In another case, N@CDs derived from bovine serum albumin reduced MRSA (ATCC 43300) by approximately 3.5 log CFU at 0.5 mg/mL, with partial inhibition at 0.125 mg/mL, indicating a dose-dependent bactericidal effect based on viable cell count [46].

The antifungal efficacy of carbon dots (CDs) exhibits significant diversity based on the precursor and synthesis method employed. SP-CDs derived from L-serine and phytic acid exhibited the highest potency, attaining minimal MIC values of 2.5 µg/mL against *Fusarium solani* and 5 µg/mL against *Candida albicans* and *Aspergillus fumigatus*, but SN-CDs demonstrated reduced efficacy at 10–20 µg/mL [39]. In contrast, glucose-derived carbon dots (CDs) demonstrated only modest inhibition, with minimum inhibitory concentration (MIC) and minimum bactericidal concentration (MBC) values of 312/625 µg/mL [40]. Amine-rich carbon dots (CDs–NH_2_) exhibited a MIC of 397 µg/mL against *Candida albicans*, while also considerably inhibiting fungal adherence (79% at 125 µg/mL), suggesting anti-biofilm properties [41]. Correspondingly, elevated MIC/MFC values for *A. fumigatus* (156/625 µg/mL) and *F. solani* (312/655 µg/mL) indicate moderate-to-weak efficacy.

Plant-derived and doped carbon dots exhibited enhanced potential. N-doped carbon dots obtained from *Trillium govanianum* successfully suppressed multidrug-resistant Candida auris, exhibiting MIC/MFC values of 0.625/1.25 mg/mL, whereas ginger peel-derived carbon dots had MIC/MBC values of 0.29/0.58 mg/mL. Nitrogen-doped carbon quantum dots derived from citric acid and urea exhibited minimum inhibitory concentration (MIC) values of 52 µg/mL and minimum fungicidal concentration (MFC) of 104 µg/mL against *Mucor indicus*, *Candida albicans*, and *Aspergillus flavus*. Conversely, CQDs@AgNPs derived from watermelon peel had significantly lower efficacy, necessitating a concentration of 15 mg/mL to inhibit *C. albicans* [62]. While the findings for [72,73] are less comprehensive, the overarching trend indicates that SP-CDs [39] exhibit remarkable potency, whereas other CDs demonstrate varying degrees of efficacy, ranging from moderate to weak, contingent upon the carbon source, surface functionalization, and doping approach [39,40,41,59,60,62,72,73,78].

## 4. Fluorescence-Based Applications of CDs in Microbial Detection

**Figure 1:**
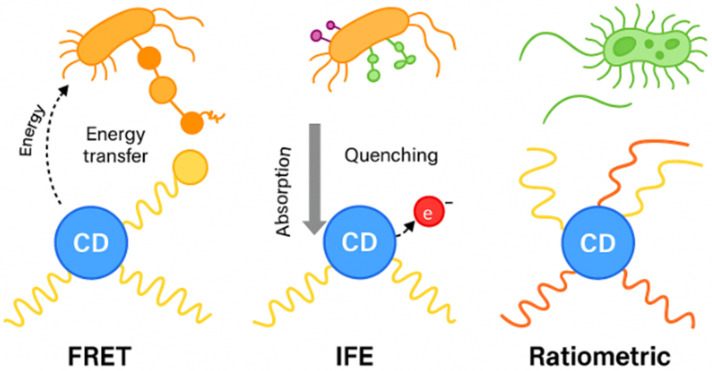
Fluorescence based applications of CDs in microbial detection. CDs (blue nanodots) can detect microbes through diverse mechanisms.

Microbial contamination remains a critical global challenge in public health and food safety, necessitating rapid and reliable diagnostic platforms. Conventional methods, such as culture plating and polymerase chain reaction (PCR), are accurate but time consuming and labor intensive. Fluorescence-based nanomaterials have become an indispensable component of modern microbial diagnostics due to their sensitivity, rapid response, and ability to operate in complex biological environments [83]. CDs have emerged as a versatile class of fluorescent nanomaterials, offering remarkable photostability, water solubility, and ease of functionalization. In microbial detection, CDs have been exploited as sensing probes due to their tunable photoluminescence, low cytotoxicity, and compatibility with biological matrices. Rapid progress has been made in the development of fluorescence-based CD biosensors for bacteria and fungi, incorporating diverse mechanisms such as Förster resonance energy transfer (FRET), inner filter effect (IFE), ratiometric (Figure 1), and photoinduced electron transfer (PET) fluorescence. These systems have been integrated with recognition elements such as antibiotics, aptamers, and boronic acids, enabling species-specific or broad-spectrum microbial detection. Applications span food safety, environmental monitoring, and clinical diagnostics.

In FRET-based systems, CDs act as fluorescent donors, transferring energy non-radiatively to an acceptor molecule or nanomaterial if the donor–acceptor distance is within 1–10 nm and there is sufficient spectral overlap. Microbial recognition events alter the spatial relationship between CDs and quenchers, leading to changes in fluorescence intensity [84]. The IFE occurs when the excitation or emission light of CDs is absorbed by nearby chromophores or nanoparticles, leading to apparent fluorescence quenching. In microbial detection, this effect has been harnessed in competitive assays, where microbial targets disrupt the absorber fluorophore interaction [85]. The advantage of IFE-based detection lies in its simplicity; no direct electron or energy transfer is required yet. It is highly sensitive to overlapping absorption spectra and solution turbidity, which must be controlled for reliable operation in food and clinical matrices [86]. Ratiometric fluorescence is an advanced detection strategy where CDs emit at two wavelengths, one responsive to microbial interactions and another serving as an internal reference. The ratiometric approach is especially valuable in real-world samples, where environmental interference often compromises single-emission assays [87]. PET mechanisms involve electron transfer between CDs and electron donors/acceptors in their environment, altering the emissive state of the CDs. Microbial metabolites, such as redox-active species, can modulate this electron transfer at the CD surface, leading to fluorescence quenching or enhancement. Additionally, functionalization of CDs with electron-rich ligands (e.g., phenolic or amine groups) has allowed PET interactions with microbial metabolites, offering selective fluorescence switching [88]. PET-based sensing is particularly useful for detecting enzymatic or metabolic activity, providing functional readouts that go beyond structural recognition of microbial surfaces. Reported CDs based fluorescent microbial assays could be seen in Table 2.

## 5. Conclusions

Carbon dots (CDs) are recognized as multifunctional nanomaterials with significant potential in addressing microbial threats. The antimicrobial activity is derived from various mechanisms, such as the generation of reactive oxygen species, disruption of membranes, and inhibition of biofilms. Effectiveness is affected by the type of precursor, heteroatom doping, and surface chemistry. Simultaneously, the intrinsic fluorescence and surface functionalization of carbon dots (CDs) render them effective tools for microbial detection, facilitating rapid, sensitive, and real-time monitoring that addresses the limitations of traditional culture-based methods. This dual role underscores the theranostic potential of CDs, integrating diagnosis and treatment within a unified framework. Challenges persist in the standardization of antimicrobial assays, enhancement of detection selectivity, and the assurance of consistent large-scale synthesis with minimal toxicity. The future development of CDs should focus on the integration of their antimicrobial and fluorescence properties into practical applications, including biosensors, point-of-care devices, and smart antimicrobial coatings. Combining pathogen detection with targeted inactivation, CDs offer an advanced strategy for managing infectious diseases and maintaining microbial safety in biomedical, food, and environmental domains.

## Figures and Tables

**Table 1:** Antimicrobial activity of CDs from the literature.

Source	Carbon Dots	Synthesis Method	Antimicrobial Methods	Microorganisms	Results	References
L-serine + phytic acid	SP-CDs and SN-CDs	Stage-melting method; nitrogen-doped	Broth microdilution (CLSI M38-A2)	*Fusarium solani*—clinical isolate	MIC SP-CDs = 2.5 µg/mL; MIC SN-CDs = 10 µg/mL	[39]
				*Candida albicans*—ATCC 10231	MIC SP-CDs = 5.0 µg/mL; MIC SN-CDs = 20 µg/mL	
				*Aspergillus fumigatus*—ATCC 204305	MIC SP-CDs = 5.0 µg/mL; MIC SN-CDs = 20 µg/mL	
Glucose	GCD (Glucose Carbon Dots)	One-step hydrothermal method	Broth microdilution (CLSI)	*Listeria monocytogenes*—ATCC 19115	MIC/MBC = 312/625 µg/mL	[40]
				*Escherichia coli*—ATCC 25922	MIC/MBC = 625/1250 µg/mL	
	BGCD (Boron-doped, boric acid)			*Listeria monocytogenes*—ATCC 19115	MIC/MBC = 312/655 µg/mL	
				*Escherichia coli*—ATCC 25922	MIC/MBC = 156/312 µg/mL	
				*Aspergillus fumigatus*—ATCC 204305	MIC/MFC = 156/625 µg/mL	
				*Fusarium solani*—clinical isolate	MIC/MFC = 312/655 µg/mL	
				*Penicillium citrinum*—ATCC 9849	MIC/MFC = 156/312 µg/mL	
				*Candida albicans*—ATCC 10231	MIC/MFC = 312/655 µg/mL	
				*Rhodotorula rubra*—ATCC 9312	MIC/MFC = 156/625 µg/mL	
	SGCD (Sulfur-doped, sodium persulfate)			*Listeria monocytogenes*—ATCC 19115	MIC/MBC = 19/78 µg/mL	
				*Escherichia coli*—ATCC 25922	MIC/MBC = 156/312 µg/mL	
				*Aspergillus fumigatus*—ATCC 204305	MIC/MFC = 312/625 µg/mL	
				*Fusarium solani*—clinical isolate	MIC/MFC = 54/108 µg/mL	
				*Penicillium citrinum*—ATCC 9849	MIC/MFC = 156/312 µg/mL	
				*Candida albicans*—ATCC 10231	MIC/MFC = 78/152 µg/mL	
				*Rhodotorula rubra*—ATCC 9312	MIC/MFC = 19/27 µg/mL	
	NGCD (Nitrogen-doped, urea)			*Listeria monocytogenes*—ATCC 19115	MIC/MBC = 19/27 µg/mL	
				*Escherichia coli*—ATCC 25922	MIC/MBC = 19/156 µg/mL	
				*Aspergillus fumigatus*—ATCC 204305	MIC/MFC = 19/39 µg/mL	
				*Fusarium solani*—clinical isolate	MIC/MFC = 78/152 µg/mL	
				*Penicillium citrinum*—ATCC 9849	MIC/MFC = 19/39 µg/mL	
				*Candida albicans*—ATCC 10231	MIC/MFC = 27/54 µg/mL	
				*Rhodotorula rubra*—ATCC 9312	MIC/MFC = 19/27 µg/mL	
D-glucosamine HCl + 1,3-diaminobenzene	CDs–NH_2_ (amine-rich)	Microwave-assisted (800 W, 3 min)	Broth microdilution (CLSI M27-A3)	*Candida albicans* ATCC 10231	MIC = 397 µg/mL; Adhesion inhibition = 79% at 500 µg/mL; Biofilm inhibition (24 h) = 89%, (48 h) = 95% at 500 µg/mL; ≥70% biofilm inhibition from 125 µg/mL; Larvae survival = 90% at 5000 µg/mL, 80% at 500 µg/mL	[41]
Urea + citric acid	CDs–CO_2_H/NH_2_ (neutral/negative)	Microwave-assisted (800 W, 30 min)			No MIC at ≤500 µg/mL; Adhesion/biofilm inhibition lower than CDs–NH_2_	
D-glucose + polyacrylate sodium	CDs–CO_2_H (negative, no N-doping)	Microwave-assisted (800 W, 4 min)			No MIC at ≤500 µg/mL; Adhesion/biofilm inhibition lower than CDs–NH_2_	
Chitosan quaternary ammonium salt (HACC) + urea + ethanolamine	N-doped carbon quantum dots (N-CQDs)	One-step hydrothermal method (optimal: m(HACC):m(urea):m(ethanolamine) = 1:2.5:1.5, 180 °C, 8 h)	Agar dilution	*Coriolus versicolor*	MIC HACC = 40 mg/mL; MIC N-CQDs = 1.8 mg/mL	[42]
Citric acid + β-alanine	CA/β-alanine CDs	One-pot microwave-assisted synthesis (700 W, 3 min)	Agar well diffusion; MIC determination (0.5–10 mg/mL); light/dark incubation	*Escherichia coli* DH5α	ZOI: 11.75 ± 0.96 mm (19 mg/mL); MIC = 1 mg/mL; complete inhibition in 16 h (19 mg/mL)	[43]
				*Pectobacterium carotovorum* Ecc7	18.00 ± 0.82 mm (19 mg/mL); MIC = 5 mg/mL; complete inhibition in 5–6 h	
				*Agrobacterium tumefaciens* EHA101	19.33 ± 1.15 mm (19 mg/mL); MIC = 1 mg/mL; complete inhibition in 5–6 h	
				*Agrobacterium rhizogenes* K599	20.33 ± 0.58 mm (19 mg/mL); MIC = 1 mg/mL; complete inhibition in 5–6 h	
				*Pseudomonas syringae* pv. *tomato* DC3000	28.67 ± 0.58 mm (19 mg/mL); MIC = 5 mg/mL; complete inhibition in 5–6 h	
				*Salmonella enterica* subsp. *enterica* serovar Typhimurium 13311	20.67 ± 0.58 mm (19 mg/mL); MIC = 1 mg/mL; complete inhibition in 11 h	
L-arginine + citric acid	N-doped arginine CDs (Arg CDs), Arg-Ag CDs, Arg-Cu CDs	One-pot, green microwave-assisted synthesis (2 min) for Arg CDs; in situ metal nanoparticle incorporation (Ag or Cu) via adsorption and NaBH_4_ reduction	Disc diffusion; broth microdilution	*Staphylococcus aureus* ATCC 6538	Disk diffusion: 6 ± 1 mm (Arg CDs), 24 ± 1 mm (Arg-Ag CDs), 22 ± 1 mm (Arg-Cu CDs) MIC: 6.25 mg/mL (Arg CDs), 0.062 mg/mL (Arg-Ag CDs), 0.625 mg/mL (Arg-Cu CDs) MBC: 12.5 mg/mL (Arg CDs), 0.125 mg/mL (Arg-Ag CDs), 3.125 mg/mL (Arg-Cu CDs)	[44]
				*Escherichia coli* ATCC 8739	Disk diffusion: 21 ± 2 mm (Arg CDs), 25 ± 1 mm (Arg-Ag CDs), 25 ± 1 mm (Arg-Cu CDs) MIC: 6.25 mg/mL (Arg CDs), 0.125 mg/mL (Arg-Ag CDs), 1.25 mg/mL (Arg-Cu CDs) MBC: 12.5 mg/mL (Arg CDs), 0.312 mg/mL (Arg-Ag CDs), 6.25 mg/mL (Arg-Cu CDs)	
PEG-400	CDs of PANI–CuO, PANI–TiO_2_, PANI–SiO_2_ (polymer–oxide composites)	Sonochemical decoration of CuO, TiO_2_, or SiO_2_ on PANI synthesized with CDs as initiator	CFU counting after mixing bacterial suspension with polymer composites; incubation at 37 °C with shaking	*Pseudomonas aeruginosa*—PAO1; *Klebsiella pneumoniae*—ATCC 700603	*P. aeruginosa:* PANI–CuO (1 mg/mL) & PANI–TiO_2_ (1 mg/mL) eradicated in 6 h; PANI–SiO_2_ (1 mg/mL) in 12 h; PANI (0.78 mg/mL) modest effect; pure oxides (CuO, TiO_2_, SiO_2_ at 220 µg/mL) no effect. *K. pneumoniae:* PANI–TiO_2_ (1 mg/mL) eradicated in 6 h; PANI–SiO_2_ (1 mg/mL) in 12 h; PANI–CuO (1 mg/mL) no effect; pure oxides (CuO, TiO_2_, SiO_2_ at 220 µg/mL) no effect.	[45]
Bovine serum albumin (BSA)	N-doped carbon dots (N@CDs) incorporated into polydopamine (PDA–N@CD composite)	Hydrothermal synthesis of N@CDs (BSA + NaOH, 180 °C, 6 h)	Viable cell count (CFU) after exposure in LB medium for 48 h	*Staphylococcus aureus*—ATCC 43300	0.5 mg/mL reduced MRSA CFU by ~3.5 logs; 0.125 mg/mL still significantly reduced viability	[46]
Riboflavin + ethylenediamine	Carbon Polymerized Dots/Polyurethane (CPDs/PU) composite films	Solvothermal method; swelling–shrink encapsulation into polyurethane	ISO 22196 antibacterial test under blue light (470 nm, 1 h)	*Klebsiella pneumoniae* ATCC 13883	Complete eradication (*R* = 5.45)	[47]
				*Proteus mirabilis* ATCC 14153	Complete eradication (*R* = 5.52)	
				*Salmonella enterica* ATCC 13076	Complete eradication (*R* = 4.81)	
				*Enterococcus faecalis* ATCC 29212	Complete eradication (*R* = 5.53)	
				*Enterococcus epidermidis* ATCC 12228	Complete eradication (*R* = 4.55)	
				*Shigella flexneri* ATCC 12022	~75% reduction (*R* = 2.10)	
				*Pseudomonas aeruginosa* ATCC 27853	Complete eradication (*R* = 4.95)	
				*Aspergillus niger* ATCC 16404	~99% reduction (*R* = 2.06)	
Fullerene C60	C60/PU composite films	Swelling–shrink encapsulation into polyurethane		*Klebsiella pneumoniae* ATCC 13883	Complete eradication (*R* = 5.45)	
				*Proteus mirabilis* ATCC 14153	Complete eradication (*R* = 5.52)	
				*Salmonella enterica* ATCC 13076	Complete eradication (*R* = 4.81)	
				*Enterococcus faecalis* ATCC 29212	Complete eradication (*R* = 5.53)	
				*Enterococcus epidermidis* ATCC 12228	Minimal effect (*R* = 0.11)	
				*Shigella flexneri* ATCC 12022	Complete eradication (*R* = 2.12)	
				*Pseudomonas aeruginosa* ATCC 27853	Complete eradication (*R* = 4.95)	
				*Aspergillus niger* ATCC 16404	Complete eradication (*R* = 5.47)	
Spermidine	Spermidine-capped carbon dots (S-PCDs)	Hydrothermal, 220 °C, 3 h using PEI + spermidine in water.	MIC in 96-well microplate; Oxford-cup inhibition zone; growth curve (OD600), spread-plate CFU and time–kill assays.	*Staphylococcus aureus* (lab isolate)	MIC = 16 µg/mL; inhibition zone 22.2 mm (S-PCDs) vs. 27.3 mm (kanamycin); at 2× MIC zone 25.8 mm; time–kill: 2× MIC ≈ kill by 8 h, 4× MIC ≈ kill by 6 h.	[48]
Phloroglucinol (1,3,5-trihydroxybenzene)	PHL-CQDs	One-step solvothermal method (ethanol + H_2_SO_4_, 180 °C, 12 h)	ISO 22196:2011 antibacterial activity test; biofilm inhibition assay	*Staphylococcus aureus* NCTC 6571; MRSA ATCC 43300; *Enterococcus faecalis* ATCC 29212; *Pseudomonas aeruginosa* ATCC 10332; *Klebsiella pneumoniae* ATCC BAA-2146; *Listeria monocytogenes* NCTC 11994; *Escherichia coli* NCTC 9001; *Acinetobacter baumannii* ATCC 19606; biofilms of *S. aureus*, *E. coli*, *P. aeruginosa*	R (log reduction): *S. aureus* = 2; MRSA = 7.7; *E. faecalis* = 2.3; *P. aeruginosa* = 0; *K. pneumoniae* = 1.3; *L. monocytogenes* = 0; *E. coli* = 5; *A. baumannii* = 5.4. Biofilms: *S. aureus* = 100% eradication; *E. coli* = ~75% reduction; *P. aeruginosa* = no effect	[49]
Citric acid + urea	CAUR-CQDs	One-step solvothermal (bottom-up); encapsulated into polyurethane via swelling–encapsulation–shrink method	ISO 22196 (measurement of antibacterial activity on plastic surfaces)	*Staphylococcus aureus* NCTC 6571	*R* = 5.2	[50]
				MRSA ATCC 43300	*R* = 4.3	
				*Enterococcus faecalis* ATCC 29212	*R* = 4.7	
				*Pseudomonas aeruginosa* ATCC 10332	*R* = 0.06	
				*Klebsiella pneumoniae* ATCC BAA2146	*R* = 5.3	
				*Listeria monocytogenes* NCTC 11994	*R* = 0.06	
				*Escherichia coli* NCTC 9001	*R* = 0.02	
				*Acinetobacter baumannii* ATCC 19606	*R* = 4.9	
Graphite electrodes (electrochemical method + gamma irradiation)	GQD50	Electrochemical exfoliation (top-down) + gamma irradiation 50 kGy; encapsulated into polyurethane via swelling–encapsulation–shrink method		*S. aureus* NCTC 6571	*R* = 0.13	
				MRSA ATCC 43300	*R* = 0.5	
				*E. faecalis* ATCC 29212	*R* = 1.12	
				*P. aeruginosa* ATCC 10332	*R* = 0.05	
				*K. pneumoniae* ATCC BAA2146	*R* = 1.5	
				*L. monocytogenes* NCTC 11994	*R* = 1.04	
				*E. coli* NCTC 9001	*R* = 0.02	
				*A. baumannii* ATCC 19606	*R* = 0.96	
*Epigynum auritum*	Near-infrared (NIR)-CDs (*Epigynum auritum*-derived)	One-step solvothermal synthesis (branches and leaves of *Epigynum auritum* in acetone at 120 °C for 5 h)	Broth microdilution	*Staphylococcus aureus*—(strain not specified)	MIC = 8 µg/mL; MBC = 32 µg/mL; growth curves show complete inhibition at 2× MIC; μmax reduced from 0.29 h^−1^ (control) to 0 h^−1^ at 16 µg/mL	[51]
				*Escherichia coli*—(strain not specified)	MIC = 62 µg/mL; MBC = 124 µg/mL; growth curves show complete inhibition at 2× MIC; μmax reduced from 0.52 h^−1^ (control) to 0 h^−1^ at 124 µg/mL	
Lignocellulose	Lignocellulose-based CQDs	One-step hydrothermal synthesis (220 °C, 12 h, 10 g/L)	Viable cell count after 24 h incubation	*Escherichia coli* ATCC 8739	0.5 mg/mL: 60.4 ± 4.1% reduction (3.9 ± 0.2 × 10^7^ CFU/mL); ≥0.75 mg/mL: >99.9% inhibition (no viable bacteria)	[52]
Polyethilenimine + citric acid	Low-C (~5–6% nanocarbon)	Microwave-assisted thermal carbonization (300 W, 4 min)	Visible-light-activated microtiter plate assay; LED light 1 h; viability by plating on LB agar	*Listeria monocytogenes* 10403S	Minimal reduction in viable cells at equivalent concentration to high-C	[53]
				*Bacillus subtilis* (laboratory model strain)	Significant inactivation under light; more susceptible than *L. monocytogenes*	
	High-C (~35–37% nanocarbon)	Microwave-assisted thermal carbonization (700 W, 4 min)		*Listeria monocytogenes* 10403S	Up to ~2 log reduction in viable cells at higher concentration	
				*Bacillus subtilis* (laboratory model strain)	Effective inactivation under light exposure	
Avocado peel	CQDs (CPC fractions)	Hydrothermal method (250 °C, 6 h)	Disk diffusion; MIC/MBC assays	*Listeria monocytogenes* ATCC 7644	Inhibition zone = 8–13 mm; MIC = 1.25–5 mg/mL; MBC = 2.5–5 mg/mL	[54]
				*Pseudomonas putida* ATCC 12633	Inhibition zone = 7–12 mm; MIC = 2.5–5 mg/mL; MBC = 5–10 mg/mL	
				*Candida albicans* ATCC 10231	Inhibition zone = 9–14 mm; MIC = 2.5–5 mg/mL; MBC = 5–10 mg/mL	
			Biofilm inhibition assay	*L. monocytogenes* ATCC 7644	Biofilm inhibition = 40–80%; biofilm eradication >50% for some fractions	
				*P. putida* ATCC 12633	Biofilm inhibition = 40–80%; biofilm eradication >50% for some fractions	
*Lactobacillus acidophilus*	L-C-dots	One-step hydrothermal method (200 °C, overnight)	Broth microdilution (CLSI)	*K. pneumoniae* ATCC 9997; Clinical carbapenem-resistant *K. pneumoniae* (CRKP) isolates	MIC L-C-dots = 50 mg/mL; Sub-MIC = 25 mg/mL; L-CFS MIC = 25 mg/mL	[55]
			96-well microtiter plate biofilm inhibition assay (crystal violet staining)		Significant biofilm inhibition at MIC and sub-MIC; % inhibition higher in L-CFS (*p* < 0.003) than L-C-dots (*p* < 0.034)	
			Checkerboard assay		Synergy in 3/5 isolates (FIC ≤ 0.5), additive in 2/5 (0.51–0.53); MIC of L-C-dots reduced from 50 mg/mL to as low as 0.78 mg/mL; meropenem from 125 µg/mL to 0.97 µg/mL in synergy cases	
			qPCR (gene expression)		fimH ↓ 0.852-fold (*p* < 0.029); mrkD ↓ 1.02-fold (*p* < 0.015)	
Marshmallow leaves extract	Marshmallow-derived CDs (CDs), CDs/ZIF-8 nanocomposite	Hydrothermal synthesis of CDs from marshmallow leaves; in situ synthesis of ZIF-8 with 2-methylimidazole and Zn(NO_3_)_2_ in presence of CDs	Broth microdilution (MIC/MBC)	*Staphylococcus aureus* (Gram+) *Escherichia coli* (Gram–)	CDs: MIC = 1000 ppm, MBC > 1000 ppm ZIF-8: MIC = 250 ppm, MBC = 1000 ppm CDs/ZIF-8: MIC = 250 ppm, MBC = 1000 ppm	[56]
Citric acid + ethylenediamine	N-CQDs	Hydrothermal (200 °C, 5 h)	Agar well diffusion; MIC (broth microdilution)	*Staphylococcus aureus* PTCC112	Zone: 12 mm; MIC: 0.5 g/L	[57]
Citric acid + ethylenediamine + Co^2+^	Co-CQDs				Zone: 15 mm; MIC: >1 g/L	
Citric acid + ethylenediamine + Fe^2+^	Fe-CQDs				Zone: 11 mm; MIC: 0.5 g/L	
Citric acid + ethylenediamine + Ni^2+^	Ni-CQDs				Zone: 6 mm; MIC: >1 g/L	
Citric acid + ethylenediamine + Cu^2+^	Cu-CQDs				Zone: 10 mm; MIC: >1 g/L	
Citric acid + ethylenediamine + Zn^2+^	Zn-CQDs				Zone: 9 mm; MIC: >1 g/L	
Citric acid + ethylenediamine	N-CQDs		Agar well diffusion; MIC (broth microdilution)	*Escherichia coli* PTCC1329	Zone: 10 mm; MIC: 0.5 g/L	
Citric acid + ethylenediamine + Co^2+^	Co-CQDs				Zone: 13 mm; MIC: >1 g/L	
Citric acid + ethylenediamine + Fe^2+^	Fe-CQDs				Zone: 11 mm; MIC: 0.5 g/L	
Citric acid + ethylenediamine + Ni^2+^	Ni-CQDs				Zone: 5 mm; MIC: >1 g/L	
Citric acid + ethylenediamine + Cu^2+^	Cu-CQDs				Zone: 9 mm; MIC: >1 g/L	
Citric acid + ethylenediamine + Zn^2+^	Zn-CQDs				Zone: 8 mm; MIC: >1 g/L	
Garlic cloves	DSCDs (Garlic-derived carbon dots)	One-step hydrothermal (50 mL garlic juice, 200 °C, 4 h; filtered, dialyzed, freeze-dried)	MIC determination (broth microdilution)	*Escherichia coli* (generic), *Staphylococcus aureus* (generic), *Pseudomonas aeruginosa* (generic)	MIC: E. coli = 32 µg/mL, S. aureus = 64 µg/mL, P. aeruginosa = 128 µg/mL; Concentration-dependent bactericidal effect (300–2000 µg/mL)	[58]
*Trillium govanianum* rhizomes	N-doped C-dots	One-step thermal calcination at 300 °C, followed by purification (centrifugation + dialysis)	Micro-broth dilution (resazurin assay) for MIC and MFC; Crystal violet biofilm inhibition assay; Bandage/Band-aid coating assay	*Candida auris* NCCPF-470197, NCCPF-470200, NCCPF-470203	MIC = 0.625 mg/mL (all strains); MFC = 1.25 mg/mL (all strains); Biofilm inhibition highest in NCCPF-470203, followed by NCCPF-470197 and NCCPF-470200; C-dots coated band-aids showed higher antifungal activity than coated bandages	[59]
Ginger rhizome peels	GiCD	Hydrothermal carbonization (200 °C, 6 h)	MIC and MBC assays (96-well microplate)	*Listeria monocytogenes*	MIC: 0.29 mg/mL; MBC: 0.58 mg/mL	[60]
				*Staphylococcus aureus*	MIC: 0.29 mg/mL; MBC: 0.58 mg/mL	
				*Escherichia coli*	MIC: 0.58 mg/mL; MBC: 1.16 mg/mL	
				*Shigella sonnei*	MIC: 0.58 mg/mL; MBC: 1.16 mg/mL	
				*Pseudomonas aeruginosa*	MIC: 0.58 mg/mL; MBC: 1.16 mg/mL	
				*Shewanella putrefaciens*	MIC: 0.58 mg/mL; MBC: 1.16 mg/mL	
			Antifungal assay (colony growth inhibition on agar)	*Aspergillus parasiticus*	Strongest inhibition among CDs; dose-dependent reduction, greatest at 3000 ppm	
				*Aspergillus flavus*	~33% suppression at 3000 ppm	
Galangal rhizome peels	GaCD	Hydrothermal carbonization (200 °C, 6 h)	MIC and MBC assays (96-well microplate)	*Listeria monocytogenes*	MIC: 1.08 mg/mL; MBC: 2.16 mg/mL	
				*Staphylococcus aureus*	MIC: 1.08 mg/mL; MBC: 2.16 mg/mL	
				*Escherichia coli*	MIC: 2.16 mg/mL; MBC: 4.32 mg/mL	
				*Shigella sonnei*	MIC: 2.16 mg/mL; MBC: 4.32 mg/mL	
				*Pseudomonas aeruginosa*	MIC: 2.16 mg/mL; MBC: 4.32 mg/mL	
				*Shewanella putrefaciens*	MIC: 2.16 mg/mL; MBC: 4.32 mg/mL	
			Antifungal assay	*Aspergillus parasiticus*	Inhibition observed but less than GiCD; effect plateaued across concentrations	
				*Aspergillus flavus*	~33% suppression at 3000 ppm	
Turmeric rhizome peels	TuCD	Hydrothermal carbonization (200 °C, 6 h)	MIC and MBC assays (96-well microplate)	*Listeria monocytogenes*	MIC: 0.59 mg/mL; MBC: 1.19 mg/mL	
				*Staphylococcus aureus*	MIC: 0.59 mg/mL; MBC: 1.19 mg/mL	
				*Escherichia coli*	MIC: 1.19 mg/mL; MBC: 2.37 mg/mL	
				*Shigella sonnei*	MIC: 1.19 mg/mL; MBC: 2.37 mg/mL	
				*Pseudomonas aeruginosa*	MIC: 1.19 mg/mL; MBC: 2.37 mg/mL	
				*Shewanella putrefaciens*	MIC: 1.19 mg/mL; MBC: 2.37 mg/mL	
			Antifungal assay	*Aspergillus parasiticus*	Moderate inhibition; less than GiCD	
				*Aspergillus flavus*	~33% suppression at 3000 ppm	
*Ginkgo biloba* leaves	GLCDs (Ginkgo Leaf Carbon Dots), nitrogen self-doped	One-step solvothermal method (180 °C, 3 h) + extraction	MIC assay (turbidimetric, 96-well); Bactericidal kinetics; ONPG assay; Nucleic acid leakage; ROS detection (DCFH-DA); SOD and CAT enzyme inhibition; TTC assay; Cytochrome oxidase inhibition; Biofilm disruption assay (crystal violet, SEM, optical microscopy)	*Staphylococcus aureus* (MRSA) ATCC 43300	MIC = 250 µg/mL; Complete eradication at 750 µg/mL in 240 min; Dose-dependent biofilm inhibition (98% destruction at 512 µg/mL); Increased membrane permeability (ONPG), nucleic acid leakage, ROS generation; Inhibition of SOD, CAT, and cytochrome oxidase activity (~80% at >350 µg/mL); Stronger biofilm destruction than vancomycin; High biocompatibility (low cytotoxicity, minimal hemolysis)	[61]
Watermelon peel	CQDs@AgNPs nanocomposites	Green hydrothermal synthesis of CQDs from dried watermelon peel + chemical reduction in AgNPs	Disk diffusion; MIC and MBC via microtiter plate method with MTT assay	*Candida albicans* (—), *Escherichia coli* (ATCC/PHTM), *Staphylococcus aureus* (ATCC/PHTM), *Acinetobacter baumannii* (ATCC/PHTM), *Klebsiella pneumoniae* (ATCC/PHTM)	*Candida albicans*—MIC 15 mg/mL, MBC 15 mg/mL (bacteriostatic); *Escherichia coli*—MIC 0.117 mg/mL, MBC 0.117 mg/mL (bactericidal); *Staphylococcus aureus*—MIC 3.75 mg/mL, MBC 3.75 mg/mL (bacteriostatic); *Acinetobacter baumannii*—MIC 3.75 mg/mL, MBC 3.75 mg/mL (bactericidal); and *Klebsiella pneumoniae*—MIC 3.75 mg/mL, MBC 3.75 mg/mL (bactericidal).	[62]
*Centella asiatica* hairy roots (transgenic line carrying *Arabidopsis thaliana* squalene synthase gene—At-SS1cDNA)	Carbon quantum dots (CQDs)	One-pot green hydrothermal method (190 °C, 10 h)	Disk diffusion and broth microdilution (MIC)	*Escherichia coli* ATCC 25922, *Staphylococcus aureus* ATCC 25923, *Pseudomonas aeruginosa* ATCC 27853	MIC: *S. aureus* = 0.25 mg/mL, *P. aeruginosa* = 1 mg/mL, *E. coli* = 2 mg/mL; Zone of inhibition: *S. aureus* = 18–22 mm, *P. aeruginosa* = 16–18 mm, *E. coli* = 11–13 mm	[63]
Curcumin + Na_2_EDTA	E-CDs	One-step hydrothermal method (180 °C, 6 h)	Broth microdilution, plate counting, disc diffusion, biofilm assay (CVS)	*Staphylococcus aureus*—ATCC 25923	MIC = 40 ppm; Plate counting: no colonies after treatment; Disc diffusion (300 µg) = 22 mm; Biofilm inhibition = 83.4% (remaining biomass 16.6%)	[64]
				*Escherichia coli*—ATCC 25922	MIC = 150 ppm; Plate counting: no colonies after treatment; Disc diffusion (300 µg) < 22 mm (smaller than *S. aureus*); Biofilm inhibition > 60% (remaining biomass < 40%);	
Tangerine peel	CQDs	Hydrothermal carbonization (190 °C, 7 h)	MIC/MBC determination; time–kill assay; tofu preservation test	*Bacillus cereus* (diarrheal type, ATCC 11778; emetic type, NCCP 14796)	MIC/MBC (µg/mL): diarrheal type = 1400/1600; emetic type = 1800/2400. CQDs at 2400 µg/mL in tofu soaking water killed diarrheal strain within 9 h and emetic strain within 12 h. Greater resistance in emetic strain. CQDs delayed growth (extended lag time) and reduced maximum population density, especially at 4 °C and 10 °C.	[65]
Glucose	CDG, ZnCDG	Hydrothermal (200 °C, 8 h)	Colony counting; well diffusion; MIC/MBC	*Staphylococcus aureus* ATCC 29213; *Escherichia coli* ATCC 35218	ZnCDG showed highest activity: complete kill at 2.5–5 mg/mL, inhibition zones 38 mm (*S. aureus*) and 32 mm (*E. coli*), MIC/MBC = 0.3125/0.625 mg/mL (*S. aureus*), 0.625/1.25 mg/mL (*E. coli*). Zinc doping improved activity over undoped CDG.	[66]
*Calendula officinalis*	CDC, ZnCDC	Hydrothermal (180 °C, 24 h)	Colony counting; well diffusion	*S. aureus* ATCC 29213; *E. coli* ATCC 35218	ZnCDC showed complete kill at 5 mg/mL, smaller inhibition zones than ZnCDG. Antimicrobial trend: ZnCDG > ZnCDC > CDG > CDC. Zinc doping plus smaller particle size correlated with higher activity.	
Rhodiola (*Rhodiola* spp.)	R-CDs	One-pot hydrothermal method (180 °C, 8 h)	MTT staining-based MIC assay (96-well plate, 12 h incubation)	*Escherichia coli* JM109 (Gram–) *Staphylococcus aureus* ATCC 6538 (Gram+)	MIC = 0.7 mg/mL (both)	[67]
Banana peel + silymarin	SL-CQDs	Hydrothermal method (200 °C, 24 h)	Agar well diffusion; broth microdilution (resazurin)	*Bacillus subtilis*; *Staphylococcus aureus*; *Escherichia coli*; *Pseudomonas aeruginosa*	MIC = 2 µg/mL for all tested strains; Zone of inhibition (100 µg/mL): *B. subtilis* = 6 mm; *S. aureus* = 7 mm; *E. coli* = 9 mm; *P. aeruginosa* = 10 mm	[68]
Keratin (human hair)	P-doped Carbon Quantum Dots (P-CQDs)	One-step hydrothermal method (180 °C, 8 h)	Broth microdilution (96-well plate, MTT assay)	*Staphylococcus aureus* (Gram-positive) ATCC—strain not specified; *Escherichia coli* (Gram-negative) ATCC—strain not specified	MIC: 0.19 mg/mL (*S. aureus*), 0.31 mg/mL (*E. coli*)	[69]
Citric acid + N-phenyl orthophenylenediamine	NCQDs (Nitrogen-doped Carbon Quantum Dots)	One-step hydrothermal method (180 °C, 8 h)	Agar well diffusion; Broth microdilution (MIC for S. aureus)	*Escherichia coli* BL21 DE3; *Pseudomonas aeruginosa*; *Bacillus subtilis*; *Staphylococcus aureus*	Zone of inhibition (mm, mean ± SD): E. coli = 11.33 ± 0.47; P. aeruginosa = 10.33 ± 0.47; B. subtilis = 13.33 ± 0.47; S. aureus = 14.33 ± 0.49. Most effective against *S. aureus*; MIC = 2–3 mg/mL	[70]
*Taxus baccata* extract (carbon source) + ethylenediamine (nitrogen source)	*T. baccata*–CDs	Biogenic hydrothermal synthesis at 150 °C for 6 h	Agar disk diffusion, MIC, MBC	*Staphylococcus aureus* ATCC 25923	Inhibition zones: 19 mm (10 µg), 33 mm (25 µg); MIC = 40 µg/mL; MBC = 80 µg/mL	[71]
				*Escherichia coli* ATCC 25922	Inhibition zones: 15 mm (10 µg), 23 mm (25 µg); MIC = 160 µg/mL; MBC = 640 µg/mL	
Zn(NO_3_)_2_·6H_2_O and AgNO_3_ with nitazoxanide	ZnO-QDs (7 nm), Ag NPs (67 nm) incorporated with nitazoxanide (NAZ)	Separate synthesis of ZnO-QDs and Ag NPs via precipitation	Agar-well diffusion; Biofilm inhibition (microdilution); MIC and MBC (broth microdilution); Time-kill assay	*Salmonella paratyphi*, *Escherichia coli*, *Klebsiella pneumoniae*, *Pseudomonas aeruginosa*, *Staphylococcus epidermidis*, *Staphylococcus aureus*, *Bacillus cereus*, *Bacillus subtilis*, *Candida krusei*, *C. glabrata*, *C. albicans*, *C. parapsilosis*	Agar-well diffusion: Largest zones—*B. subtilis* 35.25 mm, *S. aureus* 29.15 mm, *B. cereus* 27.17 mm, *S. epidermidis* 25.72 mm, *P. aeruginosa* 20.53 mm, *C. glabrata* 17.65 mm; moderate–low against others. Biofilm inhibition: Highest—*B. subtilis* 98.54%, *S. aureus* 97.98%, *S. epidermidis* 95.31%, *B. cereus* 93.45%, others 65–89%. MIC/MBC: Effective at 90–150 μg/mL for most pathogens; rapid kill in time-kill assay within 48 h.	[72]
Castor seeds	N-CQD1, N-CQD2	Hydrothermal method (180 °C and 220 °C, 24 h)	Agar disk diffusion; MIC (broth microdilution)	*Staphylococcus aureus*—ATCC 6538	N-CQD1: IZ = 30.0 ± 0.1 mm; MIC = 15.62 µg/mLN-CQD2: IZ = 30.0 ± 0.2 mm; MIC = 15.62 µg/mL	[73]
				Methicillin-resistant *S. aureus* (MRSA)	N-CQD1: IZ = 24.0 ± 0.1 mm; MIC = 31.25 µg/mLN-CQD2: IZ = 29.0 ± 0.2 mm; MIC = 15.62 µg/mL	
				*Escherichia coli*—ATCC 8739	N-CQD1: IZ = 25.0 ± 0.2 mm; MIC = 31.25 µg/mLN-CQD2: IZ = 27.0 ± 0.1 mm; MIC = 31.25 µg/mL	
				*Klebsiella pneumoniae*—ATCC 13883	N-CQD1: IZ = 25.0 ± 0.2 mm; MIC = 62.50 µg/mLN-CQD2: IZ = 26.0 ± 0.2 mm; MIC = 62.50 µg/mL	
				*Candida albicans*—ATCC 10221	N-CQD1: IZ = 30.0 ± 0.2 mm; MIC = 15.62 µg/mLN-CQD2: IZ = 31.0 ± 0.1 mm; MIC = 15.62 µg/mL	
*Ficus benghalensis* aerial roots	FB-CQDs	One-step hydrothermal) + citric acid;	Broth microdilution (96-well plate)	*Escherichia coli* ATCC 35218; *Staphylococcus aureus*	Tested at 12 concentrations (1:1 to 1:2028 *v*/*v*); no detectable MIC—both strains grew even at highest concentration; attributed to high carbonization temperature reducing antimicrobial activity	[74]
Amino acids (arginine, alanine, aspartic acid) and diethylenetriamine (DETA)	Arg-CDs	Hydrothermal method (210 °C, 6 h, pH = 12)	Plate countingmethod	*Escherichia coli* ATCC 25923, *Staphylococcus aureus* ATCC 29213	At 50,000 µg/mL (*E. coli*, *S. aureus*) antibacterial rate reached 100%; no MIC/MBC reported	[75]
Amino acids (alanine)	Ala-CDs	Hydrothermal method		*Escherichia coli* ATCC 25923, *Staphylococcus aureus* ATCC 29213	no MIC/MBC reported	
Amino acids (aspartic acid)	Asp-CDs	Hydrothermal method		*Escherichia coli* ATCC 25923, *Staphylococcus aureus* ATCC 29213		
Amino acids (arginine, optimized synthesis)	Arg-CDs-Op	Hydrothermal method (optimized: 210 °C, 6 h, pH = 12)	MIC/MBC assays	*E. coli* ATCC 25923, *S. aureus* ATCC 29213	MIC = 2000 µg/mL (*E. coli*), MIC = 19,000 µg/mL (*S. aureus*)	
Amino acids (arginine) + DETA	DETA-Arg-CDs	One-pot hydrothermal synthesis 210 °C, 6 h, pH = 12)	MIC/MBC assays; inhibition zone test; time-kill curve	*E. coli* ATCC 25923, *S. aureus* ATCC 29213	MIC = 8 µg/mL (*E. coli*), MIC = 4 µg/mL (*S. aureus*); MBC = 16 µg/mL (*E. coli*), MBC = 8 µg/mL (*S. aureus*); killed > 90% *E. coli* in 5 min and *S. aureus* in 2 min; inhibition zone radius: 1.5 cm (*E. coli*), 1.75 cm (*S. aureus*)	
*Prosopis juliflora* leaves	PJ-CDs	Hydrothermal method	Agar well diffusion (zone of inhibition), broth microdilution	*Staphylococcus aureus* ATCC 25923; *Escherichia coli* ATCC 25923	MIC (*S. aureus*) = 1.50 mg/mL; Zone of inhibition (*S. aureus*) = 18 mm; No inhibition against *E. coli*	[76]
Ammonium citrate	CDs conjugated to thiolated-ureido-chitosan/PMLA nanoparticles (UCPM-NPs) loaded with amoxicillin (AMX-UCPM-NPs)	Heating ammonium citrate at 180 °C, 2 h) + ionic gelation for NPs + carbodiimide coupling	Bacterial viability assay (OD_590_), LIVE/DEAD staining, membrane permeability assay (NPN uptake)	*Helicobacter pylori* ATCC 26695 (in vitro)	MIC AMX-UCPM-NPs (AMX) = 0.5 mg/mL → complete eradication; free AMX, AMX-CPM-NPs, CDs, and CDs + AMX less effective	[77]
	Blank UCPM-NPs (no AMX)		Membrane permeability assay (NPN uptake)	*H. pylori* ATCC 26695 (in vitro)	Strong membrane permeabilization (↑RFU) via ROS and pore formation; confirmed by TEM/SEM	
	AMX-UCPM-NPs		Viability assay in mucus barrier model	*H. pylori* ATCC 26695 (in vitro, mucus layer)	91.5% viability reduction despite mucus; higher efficacy than AMX-CPM-NPs	
	AMX-UCPM-NPs		In vivo infection model (oral gavage, 7 days)	*H. pylori* SS1 (mouse model)	7.5 mg/kg/day AMX dose → effective eradication, normal gastric histology, reduced ulcers	
Citric acid (carbon source) + urea (nitrogen source)	Nitrogen-doped carbon quantum dots (N/CQDs)	Microwave-assisted synthesis (2:1 mass ratio citric acid:urea, 450 W, 10 min, dialysis)	MIC, MFC, sorbitol assay, agar diffusion, antifungal inhibition %, in vivo wound healing and CFU count	*Mucor indicus* CNRMA 03894, *Candida albicans* RCMB 05035, *Aspergillus flavus* RCMB 02782, *A. fumigatus* RCMB 02564, *A. niger* RCMB 02568, *Penicillium notatum* NCPF 2881	MIC (µg/mL): *M. indicus* 52, *C. albicans* 52, *A. flavus* 208, *A. fumigatus* 333, *A. niger* 208, *P. notatum* 52; MFC (µg/mL): *M. indicus* 3, *C. albicans* 4, *P. notatum* 4; Sorbitol MIC: *M. indicus* 8, *C. albicans* 10, *P. notatum* 13; Inhibition %: *M. indicus* 98%, *C. albicans* 83%; Significant wound healing and CFU reduction *in vivo*	[78]
Phenol + melamine + formaldehyde	Nitrogen-doped mesoporous carbon (N/MC)	Hydrothermal synthesis (130 °C, 12 h) + drying (60 °C, 12 h)			MIC (µg/mL): *M. indicus* 52, *C. albicans* 250, *A. flavus* 250, *A. fumigatus* 500, *A. niger* 250, *P. notatum* 250; MFC (µg/mL): *M. indicus* 2, *P. notatum* 14; Sorbitol MIC: *M. indicus* 26; Inhibition %: *M. indicus* 98%, *C. albicans* 83%; Significant wound healing and CFU reduction *in vivo* (less effective than N/CQDs for fungal clearance)	
L-arginine	CDs-Arg and CDs-P (HPMC-modified CDs-Arg)	Hydrothermal synthesis at 200 °C for 4 h; modification with hydroxypropyl methylcellulose (7.5%, 15%, 30%)	Broth dilution MIC determination; Disk diffusion (inhibition zone)	*Staphylococcus aureus*—ATCC 6538	MIC = 1.25% (both CDs-Arg and CDs-P); Inhibition zone: CDs-Arg = 2.2 mm (5%), 2.1 mm (2.5%), 1.4 mm (1.25%); CDs-P = 2.0 mm (5%), 1.9 mm (2.5%), 1.0 mm (1.25%)	[79]
				*Escherichia coli*—ATCC 8099	MIC = 1.25% (both CDs-Arg and CDs-P); Inhibition zone: CDs-Arg = 2.0 mm (5%), 1.1 mm (2.5%), 1.0 mm (1.25%); CDs-P = 1.8 mm (5%), 1.4 mm (2.5%), 0.9 mm (1.25%)	

**Table 2:** Comparison of reported CDs based fluorescent microbial assays.

Target (Matrix)	Probe Design and Mechanism	Read Out Time	Linear Range/LOD	Notable Features	References
Gram-positive bacteria (buffer)	Vancomycin-CDs. Surface binding with turn-on imaging	<30 min	Imaging (qualitative), LOD reported for cell counts in follow-ups	Classic antibiotic guided gram typing with CDs	[89]
*E. coli* (culture/imagery)	Aptamer functionalized Graphene QDs (GQDs), binding with fluorescence and AI image analysis	30–60 min	Correlated intensity and CFU (image based)	Demonstrates coupling to AI for quantitative counting	[90]
*E. coli O157:H7* (foods)	CD-COF PRET sensor, target disrupts FRET	≤30 min	Sub-100 CFU mL^−1^, wide linear range (report specific)	High sensitivity, food matric compatible	[91]
Mixed bacteria (paper device)	Paper sensor array of functionalized CDs + ML classifier	Minutes	Species level discrimination, confusion matrix accuracy reported	Portable, label pattern recognition	[92]
*E. coli* (water/food)	CQD-Aptamer + AgNP IFE/FRET platform, target restores CD emission	30 min	nM-pM (oligo surrogates) or CFU mL^−1^, low LODs	Green CQDs synthesis, robust IFE/FRET switching	[93]
*Candida albicans* (biofluids)	N-CQDs turn on assay. Surface interaction enhances emission	<30 min	nM-µM labels/CFU mL^−1^ detection (report-specific)	Rapid fungal sensing, good biocompatibility	[94]
*Vibrio parahaemolyticus* (foods)	Xylan derived ratiometric CD platform (dual band)	≤40 min	Ratiometric calibration, low LOD (report specific)	Matrix tolerant quantitation via internal referencing	[95]

Notes: Exact numerical LODs/linear ranges vary by platform and matrix, ML = machine learning, CFU = colony forming units.

## Data Availability

All data presented in this review are from previously published studies, which have been appropriately cited. No new data collection involving humans or animals was conducted.
